# The economic imperative for an AI Productivity Index in healthcare: Beyond clinical promise to fiscal accountability

**DOI:** 10.1016/j.clinme.2026.100638

**Published:** 2026-08-20

**Authors:** Yazan A. Al-Ajlouni, Basile Njei

**Affiliations:** a Montefiore Medical Center/Einstein School of Medicine, Bronx, NY, United States; b Yale International Medicine Program, Yale University, New Haven, CT, United States; c Section of Digestive Diseases, Department of Medicine, Yale University, New Haven, CT, United States; d Artificial Intelligence Programme, University of Cumbria, Carlisle, United Kingdom; e Ohio University Heritage College of Osteopathic Medicine, Athens, OH, United States; f Yale Liver Center, Yale New Haven Health, New Haven, CT, United States; g Engelhardt School of Global Health and Bioethics, Euclid University, Bangui, Central African Republic

**Keywords:** Artificial intelligence, Healthcare productivity, Health system efficiency, Digital health evaluation, Health technology assessment, Clinical workflow

## Abstract

Artificial intelligence (AI) is increasingly embedded in healthcare delivery, yet its evaluation remains dominated by technical performance metrics that inadequately capture real-world system value. Clinical accuracy is necessary but insufficient to justify adoption in resource-constrained health systems facing workforce shortages and rising demand. AI technologies compete for limited financial, technical and cognitive resources, but there is no standardised framework to assess their effects on productivity, workflow, downstream utilisation or equity. This article argues that treating clinical validity as a proxy for value risks misallocating scarce resources and undermining trust in digital transformation. We propose an AI Productivity Index to complement existing safety, efficacy and economic assessments by evaluating operational impact, implementation burden, opportunity costs and post-deployment consequences. Embedding productivity measurement into procurement and governance processes could help align AI innovation with fiscal accountability, equitable access and sustainable healthcare delivery.

Artificial intelligence (AI) has rapidly moved from experimental promise to routine deployment across healthcare systems, supporting diagnostic interpretation, clinical decision support, and administrative workflows.^1^ This expansion has been driven primarily by demonstrations of technical feasibility and increasing attention to ethical considerations such as fairness, transparency, and patient safety. Yet this dominant narrative has evolved with limited engagement with the economic constraints of healthcare delivery. Health systems operate under persistent fiscal constraints, workforce shortages, and rising demand for care, making AI not merely a clinical innovation but an economic intervention competing for scarce resources.^2^ Despite this, there remains no standardised framework to assess whether AI adoption meaningfully improves productivity or resource utilisation in healthcare. The absence of such accountability risks allowing investment decisions to proceed based on promise rather than demonstrable system value, threatening the financial sustainability of digital transformation efforts.

The evaluation of healthcare AI remains overwhelmingly anchored in clinical performance metrics such as sensitivity, specificity, and discrimination, reinforcing the assumption that technical accuracy equates to real-world value. While clinical validity is a necessary condition for safe deployment, it is insufficient to determine system impact. AI tools are introduced into complex clinical environments where even modest increases in cognitive burden, documentation time, or workflow disruption can offset theoretical gains in accuracy. Moreover, AI implementation entails substantial opportunity costs: financial investment, information technology capacity, and clinician attention devoted to one system are resources unavailable for alternative interventions.^3^ As a result, highly accurate tools with low real-world adoption or poor integration may generate negative net productivity, consuming resources without improving efficiency or throughput. Treating clinical validity as a proxy for system value represents a fundamental conceptual error that obscures the true economic consequences of AI adoption.

This distinction is not merely theoretical. In an external validation study of a widely implemented proprietary sepsis prediction model, Wong *et al.* found poor discrimination and calibration, raising concerns about broad deployment without local validation and continuous monitoring.^4^ The example is instructive because prediction tools do not generate value through discrimination alone. Their operational value depends on alert burden, clinician response, workflow integration, and whether downstream actions improve care without consuming disproportionate clinical resources. A technically promising model may therefore create limited or even negative net productivity if it increases alerts, verification work, or low-yield clinical activity.

Correcting this error requires evaluation frameworks that capture how AI technologies interact with real-world workflows and constrained health system resources. Existing approaches establish technical performance and safety but rarely measure determinants of productivity, including decision latency, workflow disruption, integration burden, and downstream utilisation. Usability and acceptability studies are also insufficient, as clinician satisfaction does not necessarily translate into improved throughput or efficiency and may obscure downstream effects such as increased investigations, documentation burden, or referral cascades.^5^ Traditional health economic analyses remain valuable, but are often too slow and model-dependent to guide decisions in a rapidly evolving AI landscape.

The absence of standardised productivity assessment also shapes the healthcare AI market. Health systems often procure AI tools under information asymmetry, relying on vendor-reported performance metrics and marketing claims about efficiency gains without independent verification.^6^, ^7^ In the absence of comparable benchmarks, procurement may be driven by perceived innovation, regulatory clearance, or promotional sophistication rather than demonstrated system-level value.

This resembles earlier pharmaceutical markets before widespread health technology assessment, when pricing and diffusion were weakly linked to value.^8^ In the AI ecosystem, vendors have limited incentives to optimise for workflow integration, adoption, or downstream efficiency when success is measured primarily by technical performance and sales.^9^ A shared productivity framework would reduce information asymmetry, allow meaningful comparison across technologies, and shift competition from claims to evidence of system benefit.

An AI Productivity Index should begin with the premise that productivity in healthcare cannot be reduced to a single outcome or performance indicator. AI systems operate within complex clinical environments, where gains in speed, accuracy, or automation may be offset by added cognitive burden, workflow disruption, or downstream resource use.^10^, ^11^ Any meaningful assessment must therefore treat productivity as a composite construct that reflects how AI technologies function in practice, rather than how they perform under idealised conditions. This requires moving beyond narrow efficiency claims toward an integrated view of system impact.

To make this concept operational, the AI Productivity Index should be understood as a preliminary composite framework rather than a single fixed metric. Its purpose would be to provide a structured, transparent assessment of whether an AI tool improves the functioning of a clinical service after accounting for workflow effects, implementation burden, downstream resource use, and equity. The domains proposed in Box 1 are illustrative rather than definitive, but they demonstrate how productivity could be assessed in a way that is measurable, comparable, and relevant to health system decision-making.

Box 1 **Illustrative domains for an AI Productivity Index in healthcare.**

**Table:** 

Domain	What the domain captures	Potential measures
Clinical workflow efficiency	Whether the AI tool reduces time, delay, or operational friction in routine care	Time to decision; reporting or documentation time; patient throughput; length of task completion; turnaround time
Integration and implementation burden	The resources required to deploy, maintain, and safely operate the tool	IT support requirements; training time; EHR integration burden; implementation cost; frequency of technical interruptions; need for human workarounds
Clinician cognitive burden and adoption	Whether the tool supports clinical judgement or adds alert fatigue, verification burden, or distrust	Override rates; alert volume; clinician-reported cognitive load; adoption and sustained use; time spent verifying AI outputs
Downstream resource utilisation	Whether the tool changes subsequent use of healthcare resources in beneficial or unintended ways	Additional tests; referrals; admissions; follow-up appointments; false-positive cascades; avoidable investigations
Equity and access impact	Whether productivity gains are distributed across populations and settings rather than concentrated in well-resourced environments	Adoption, performance, time savings, alert burden, and downstream utilisation stratified by site, deprivation, demographic group, language, disability, and digital access; effect on waiting times or access for underserved groups

Importantly, the Index should not replace clinical validation, safety assessment, or formal economic evaluation. Rather, it would complement these approaches by asking whether an AI tool improves the productive capacity of a real clinical service once its full operational consequences are considered. This includes workflow efficiency, implementation burden, downstream resource use, and whether benefits and burdens are distributed equitably across sites, clinical teams, and patient groups. The value of a structured Index is that it makes trade-offs explicit: a tool that shortens one task but increases verification time, alert burden, downstream testing, or inequitable access may have limited or negative net productivity.

Within the NHS, an AI Productivity Index would sit alongside existing assurance mechanisms rather than replace them. NICE’s Evidence Standards Framework supports evidence generation for digital health technologies, while the AI and Digital Regulations Service provides guidance for developers and adopters across evaluation and regulation. NHS procurement guidance also encourages buyers to assess safety, evidence, governance, implementation and organisational fit, and MHRA regulation of software and AI as medical devices focuses on safety, performance and patient protection.^12^, ^13^, ^14^, ^15^ However, these frameworks do not fully answer the operational question of whether an AI tool improves the productive capacity of a real clinical service after implementation. A productivity index could therefore be applied before procurement to assess whether claimed efficiency gains are plausible and measurable, during early implementation to identify workflow friction and downstream effects, and after deployment to inform renewal, scaling or decommissioning. This adapts the value-based logic of HTA to AI’s rapid iteration, local workflow dependence and evolving real-world performance.

An AI Productivity Index should function not merely as an evaluative tool, but as a governance mechanism embedded within health system decision-making. In practice, this implies a graduated approach to evaluation, in which early-stage assessments inform procurement decisions and are followed by structured monitoring as technologies move into real-world deployment. Productivity measurement should not end at adoption; continuous post-implementation assessment is essential to capture how performance evolves as clinicians adapt, workflows change, inequities in benefit or burden become visible, and unintended consequences emerge. Over time, such measurement could inform reimbursement decisions, guide renewal or decommissioning of AI tools, and support policy frameworks that align innovation with demonstrable system benefit. Importantly, productivity certification would complement, not replace, existing safety and efficacy requirements, extending accountability beyond technical validity to operational value.

Concerns regarding context dependence, domain weighting, and temporal learning curves are legitimate but do not undermine the need for standardisation. Productivity will inevitably vary across settings, and rigid thresholds would be inappropriate in complex clinical environments. However, flexibility should be understood as a design feature rather than a weakness. A well-constructed index can accommodate local priorities, evolving usage patterns, and differential learning effects while still enabling transparent comparison and accountability. The greater risk lies not in over-standardisation, but in continued reliance on unstructured judgement and non-comparable evidence. Without a shared framework, health systems remain unable to distinguish between technologies that merely perform well in theory and those that deliver sustained value in practice.

Healthcare AI has reached a point of maturity where enthusiasm alone is no longer sufficient to justify continued investment. As AI becomes embedded in clinical practice, its adoption must be guided by the same principles of stewardship that govern other uses of limited healthcare resources. Productivity measurement is not a technical add-on, but an ethical obligation necessary to ensure that innovation strengthens, rather than strains, health systems operating under increasing financial and workforce pressures. Continued investment in AI technologies without standardised accountability risks eroding institutional trust, misallocating scarce resources, and ultimately undermining support for digital transformation. Conversely, tools that demonstrably enhance productivity, efficiency, and equitable access deserve scalable adoption and sustainable financing. Establishing an AI Productivity Index offers a pragmatic pathway to distinguish between promise and value. Health systems, regulators, and developers share responsibility for advancing this transition. By embedding productivity assessment into evaluation, procurement, and governance processes, stakeholders can align innovation with fiscal accountability and long-term system resilience. Measuring productivity should therefore be viewed not as a barrier to progress, but as a prerequisite for the sustainable future of healthcare AI.

## CRediT authorship contribution statement

**Yazan A. Al-Ajlouni:** Writing – review & editing, Writing – original draft, Validation, Project administration, Conceptualization. **Basile Njei:** Writing – review & editing, Writing – original draft, Validation, Supervision, Resources, Methodology, Conceptualization.

## Funding

This research did not receive any specific grant from funding agencies in the public, commercial, or not-for-profit sectors.

## Declaration of competing interest

The authors declare that they have no known competing financial interests or personal relationships that could have appeared to influence the work reported in this paper.
